# Diagnostic Performance of Competitive ELISA and Western Blot Methods for the Detection of Antibodies against *Theileria equi* and *Babesia caballi*

**DOI:** 10.3390/microorganisms11010021

**Published:** 2022-12-21

**Authors:** Guangpu Yang, Bingqian Zhou, Kewei Chen, Zhe Hu, Wei Guo, Xiaojun Wang, Cheng Du

**Affiliations:** State Key Laboratory of Veterinary Biotechnology, Harbin Veterinary Research Institute, Chinese Academy of Agricultural Sciences, Harbin 150069, China

**Keywords:** *Theileria equi*, *Babesia caballi*, competitive ELISA, western blot

## Abstract

*Theileria equi* (*T. equi*) and *Babesia caballi* (*B. caballi*) are the causative pathogens of Equine piroplasmosis (EP), a disease that has brought huge economic losses and great restrictions to the global equine industry. Rapid and accurate diagnostic methods are critical for the effective monitoring of the disease. In this study, we developed novel competitive ELISA methods and western blot assays based on the EMA1 or Bc48 proteins to detect antibodies against *T. equi* or *B. caballi*, respectively. In the novel cELISA, horseradish peroxidase (HRP)-labeled monoclonal antibodies are used in place of enzyme-conjugated secondary antibodies, in order to speed up the entire procedure. These methods have high sensitivity and no cross-reactivity with antibodies against other equine diseases. In the newly developed western blot assays, we optimized the dilution of *T. equi* or *B. caballi* positive serum samples to 1:200. Compared with the commercially available kit, both the novel cELISA assay and the western blot assay showed high coincidence rates in detecting antibodies against *T. equi* and *B. caballi*. Taken together, the novel cELISA and the western blot assays for detecting antibodies against *T. equi* or *B. caballi* have the potential to rapidly test for *T. equi* or *B. caballi* and to contribute to the surveillance and control of this disease.

## 1. Introduction

Equine piroplasmosis (EP) is an important protozoan infectious disease, caused by *Theileria equi* (*T. equi*) and *Babesia caballi* (*B. caballi*), and affects equids worldwide [1]. The clinical presentation is related to intravascular hemolysis and associated systemic illness, characterized by fever, anemia, loss of appetite, edema, jaundice, hepatomegaly, and splenomegaly [2,3,4]. The prevalence of this disease is related to many risk factors, including animal species, age, gender, breed, activities, and environmental factors [5]. EP has caused huge economic losses to the horse industry, including the cost of treatment and death. Moreover, its occurrence has caused restrictions in equine trading [6]. Because of the veterinary and economic impact of this disease, the World Organization for Animal Health (WOAH) has listed EP as a notifiable disease [7]. Many countries prevent the movement of *T. equi*- or *B. caballi*-positive animals into disease-free areas through strong restrictions [8]. This highlights the necessity of effective control measures in countries where the disease is endemic, and of enhancing the detection of EP in animals exposed to the parasites that cause it.

To date, the diagnostic methods used to detect *T. equi* and *B. caballi* infection include microscopic examination, the complement fixation technique (CFT), the indirect fluorescence antibody test (IFAT), enzyme-linked immunosorbent assay (ELISA), and PCR [9,10,11,12]. Nevertheless, in both acute and early infections, serological testing becomes an important diagnostic method for confirming the involvement of *T. equi* or *B. caballi*, and ELISA has been widely used in detecting antibodies against *T. equi* and *B. caballi* [13]. Based on the different approaches, the ELISA assay has been divided into indirect ELISA, competitive ELISA (cELISA), blocking ELISA, and others [14]. Currently, the WOAH has listed cELISA as the preferred test for EP diagnoses in the international equine trade and recommends it as the gold standard for serological testing for *T. equi*- and *B. caballi*-specific antibodies in equids [7].

The Equi Merozoite Antigen 1 (EMA1) or Bc48 is an important candidate antigen to detect antibodies against *T. equi* or *B. caballi*. Many previous works have identified EMA1 or Bc48 as an antigen recognized by most serums of *T. equi-* or *B. caballi*-infected horses. This indicated that the host has strong humoral immunity against EMA1 or Bc48. The erythrocytic-stage surface protein, EMA1 is a major candidate for the development of a diagnostic antigen for equine piroplasmosis [15]. Bc48 is one rhoptry protein of merozoites of *B. caballi*, which was previously evaluated as a promising antigen for the serological detection of antibodies to *B. caballi* [16].

cELISA kits are available commercially, but require multiple steps, including dilution of serum, incubation of primary antibody, and incubation of secondary antibody, as well as washing at each step, which have many operation steps and take a long time [17,18]. In China, there was just one sort of commercial kit available for the diagnosis of EP. In this study, we describe novel cELISA and western blot assays developed with recombinant proteins EMA1 and Bc48 and the corresponding monoclonal antibodies (mAbs), which can specifically diagnose the presence of antibodies of *T. equi* or *B. caballi* in equids. Horseradish peroxidase (HRP)-labeled mAbs were used in the novel cELISA. In addition to the removal of the necessity for secondary antibody incubation and washing, the novel cELISA approach also reduces testing time. Most epidemiological data arise from cELISA of anti-*T. equi* and anti-*B. caballi* antibodies in sera, as a high throughput of samples at comparably low cost, can be achieved with this method. However, the sera from equids suspected to be infected with either *T. equi* or *B. caballi* are typically tested for individual diagnosis using western blotting [19]. The advantage of the western blotting method is that it can detect both the conformational epitopes and the linear epitopes of the EMA1 and Bc48 antigens. In addition, western blotting constitutes a highly specific detection method that tends to be less cross-reactive with pathogens other than *T. equi* and *B. caballi*, and it is therefore used to confirm ELISA-positive results [20]. Western blot assays were thus an effective supplementary method in the diagnosis of *T. equi* or *B. caballi* infection. The novel cELISA and western blot assays developed here showed high diagnostic sensitivity and specificity for the detection of antibodies against *T. equi* or *B. caballi*. In addition, when assessing serum samples, the cELISA and western blot assays showed a high coincidence rate with the commercial cELISA kit. These developed methods provide a rapid and accurate diagnosis of EP and will help to reduce the economic impact of *T. equi* or *B. caballi* infection.

## 2. Material and Methods

### 2.1. Serum Samples

A total of 200 test serum samples were collected from Inner Mongolia, China from 2019 to 2020, and were used to determine the cut-off value of the novel cELISA and to compare it with the commercially available cELISA. A further 208 test serum samples were collected from Inner Mongolia, China in 2022, and were used for comparative analysis of the clinical samples. The serum samples positive for *Equine infectious anemia virus* (EIAV), *Equine influenza virus* (EIV), *Equine herpesvirus* (EHV), *Equine arteritis virus* (EAV), *B. caballi*, *Burkholderia mallei* (*B. mallei*), *Trypanosoma evansi* (*T. evansi*), *Escherichia coli* (*E. coli*), *Streptococcus equi* (*S. equi*), *T. equi* or *Salmonella abortus equi (S. abortus equi)* and the serum samples negative for *T. equi* and *B. caballi* were all stored in our laboratory.

### 2.2. Preparation of the Proteins EMA1 and Bc48

In order to prepare the antigens of *T. equi* and *B. caballi* for ELISA assays, the recombinant plasmids pET-30a-EMA1 and pET-32a-Bc48 were constructed and introduced into *E. coli* BL21 cells (Tsingke, Beijing, China) as described previously [21]. The EMA1 and BC48 proteins were expressed and purified by affinity chromatography on a Ni-nitrilotriacetic acid (NTA) agarose column system (Genscript, Piscatvie, NJ, USA) and analyzed with SDS-PAGE as previously described [21].

### 2.3. Generation of HRP-Conjugated mAbs against EMA1 and Bc48

mAbs against EMA1 and Bc48 proteins were generated as previously described [21]. Briefly, specific-pathogen-free 6-week-old female BALB/c mice (purchased from Liaoning Changsheng Biotechnology Co., Ltd., Shenyang, China) were immunized with 100 μg protein (either EMA1 or Bc48). Five days after the final booster, the immunized mice were subjected to hybridoma cell preparation. Supernatants from growing hybridoma cells were screened using an indirect ELISA for reactivity to EMA1 or Bc48 proteins. The positive hybridoma clones were subcloned until monoclonal cell lines were obtained. Isotypes of the generated mAbs were then determined using an SBA clonotyping system-HRP (Southern Biotech, Birmingham, Alabama, USA). The reciprocal competition was analyzed between selected mAbs and the serum samples positive for *T. equi* and *B. caballi* [21].

Ascites fluid containing mAb was prepared by injecting 5 × 10^5^ selected hybridoma cells into the abdominal cavity of each mouse. One week prior to the ascitic fluid culture, mice were injected with Freund’s incomplete adjuvant (Sigma, Ronkonkoma, NY, USA). The ascitic fluid containing the mAb was purified using a protein G perfusion affinity chromatographic column (GE Healthcare, Chicago, IL, USA). The purified mAbs were dialyzed in PBS (10 mmol/L) and determined using an UV spectrophotometer at 280 nm.

Purified mAbs were conjugated to HRP using an HRP-labeling kit (HRP Conjugation Kit-Lightning-Link^®^, Abcam, Cambridge, UK) according to the manufacturer’s instructions. Briefly, each 10 µL of antibody was given 1 µL of the modifier reagent, and they were gently mixed. After removing the cap from vial of HRP Conjugation Mix, the antibody sample was pipetted directly onto the lyophilized material, to which was added Modifier reagent. The liquid was resuspended gently by pipette, covered and left standing for 3 h in the dark at room temperature (20–25 °C). Then, 1 µL of Quencher reagent was added for each 10 µL of antibody and the mixture was gently stirred. Finally, the HRP-labeled mAbs were stored in −80 °C refrigerator with 50% glycerol.

### 2.4. Establishment of cELISA for Diagnosis of T. equi or B. caballi

To optimize the amounts of coating antigen to be used, a checkerboard assay was conducted as follows. The coating protein EMA1 was diluted to five concentrations (0.1 µg/mL, 0.5 µg/mL, 0.75 µg/mL, 1 µg/mL, 1.5 µg/mL) and Bc48 was diluted to six concentrations (0.5 µg/mL, 0.75 µg/mL, 1 µg/mL, 1.25 µg/mL, 1.5 µg/mL, 2 µg/mL). To determine the optimal dilution of the sera to be tested, four positive and four negative sera were diluted to generate four serial twofold dilutions (1:1, 1:2, 1:4, and 1:8). 5% skimmed milk, 5% BSA and 5% PBS were used to determine the optimum block buffer, and the plate was stained with 3,3′,5,5′-tetramethylbenzidine (TMB)-ELISA Substrate Solution (InnoReagents, Zhejiang, China) for different lengths of time (5 min, 10 min, 15 min, 20 min, and 25 min) to determine the optimum substrate reaction time. The optimum reaction conditions for use in this cELISA were selected based on the minimum ratio of OD_450nm_ values between positive and negative sera (P/N).

The standard cELISA procedure was as follows: a 96-well plate was coated with antigen (100 μL/well), which was in PBS buffer (0.1 M, PH 7.4) at optimum concentrations at 2–8 °C for 12–16 h. After washing three times with 200 μL PBST (PBS with 0.1% Tween), the plate was blocked with the optimum block buffer in incubator at 37 °C for two hours and washed three times with PBST again. Next, the tested sera at the optimal dilution were added to the wells (100 μL/well) and the plate was put into an incubator at 37 °C for thirty minutes. After washing again, 100 μL of diluted HRP-conjugated IgG was added to each well and mixed. Incubation of the plate was performed for 30 min at 37 °C and washed three times with 200 μL washing buffer. The plate was stained with TMB at 15–25 °C for the optimum reaction time and the reaction was stopped by the addition of 50 μL 2 M H_2_SO_4_. Finally, the OD_450nm_ value of the plate was measured using enzyme calibration (Biotek, Winooski, Vermont, USA) based on the values from the positive and negative sera.

### 2.5. Determination of the Cut-Off Value of the Novel cELISA

A total of 200 sera were collected from Inner Mongolia from 2019 to 2020 and kept in our laboratory. Of these, 47 samples were determined to be positive for *T. equi* and 25 samples were determined to be positive for *B. caballi* [21]. The serum samples were used to determine the cut-off value for the novel cELISA. The OD_450nm_ values of the samples were converted to a percent inhibition (PI) value using the following formula: PI (%) = (1 − (OD_450nm_ value of sample ÷ OD_450nm_ value of negative serum)) × 100%. In this study, we determine the baseline cut-off values by assessing the maximum consistency between the detection results of the novel cELISA methods and the identification of the serum samples.

### 2.6. Determination of the Analytical Specificity and Sensitivity of the Novel cELISA

To evaluate the analytical specificity of the novel cELISA, serum samples positive for *T. equi* and *B. caballi*, *E. coli*, *S. abortus equi*, *S. equi*, EHV-4, EAV, *B. mallei*, EIAV, and *T. evansi*, as well as serum samples negative for *T. equi* and *B. caballi*, were simultaneously tested. The analytical sensitivity of the novel cELISA was determined with a serial dilution of a separate serum sample positive for *T. equi* or *B. caballi*, which was diluted at ratios of 1:2, 1:4, 1:8, 1:16, 1:32, 1:64, 1:128, 1:256, and 1:512 with PBS. These dilutions were also used to test the lowest dilution at which the novel cELISA could still detect the diseases.

### 2.7. Comparison of the Novel cELISA and Commercially Available cELISA Kit

The novel cELISA and the commercially available cELISA kit (VMRD, Pullman, WA, USA) were used in parallel to detect antibodies against *T. equi* and *B. caballi*. The 200 serum samples that were used in the cut-off determined section above were tested in this comparative analysis. These tests of the commercial kit were conducted following the manufacturer’s instructions. We performed ROC curve analysis as a statistical tool for the diagnostic evaluation of the two cELISA assays in surveillance samples, where the value for the area under the curve (AUC) enabled a combined measure of diagnostic sensitivity and specificity (an AUC of 1 indicates perfect discriminatory value; an AUC of 0.5 or less indicates no discriminatory value).

### 2.8. Establishment of a Western Blot Assay That Detects T. equi and B. caballi Antibodies

SDS-PAGE Bis-Tris gels were prepared with PAGE Gel Fast Preparation Kit (Shanghai Epizyme Biomedical Technology Co., Ltd, Shanghai, China) for western blotting. The prepared protein EMA1 or Bc48 was separated by electrophoresis on SDS-PAGE Bis-Tris gel and run over two stages, including 80 V for 20 min and 120 V for 40 min. Next, the separated protein was transported onto nitrocellulose membranes and blocked with 5% skimmed milk (with NaN_3_) for 2 h at room temperature. Membranes were incubated with test sera for 1.5 h and washed three times for 10 min with tris-buffered saline/Tween (TBST). Then, the membranes were incubated with DyLight 680-labeled goat anti-equine secondary antibodies (1:5000 in PBS) (NOVUS, Minneapolis, MN, USA) for 40 min and washed again. Finally, the target bands were detected using an Odyssey imaging system (LI-COR, Lincoln, NE, USA). To determine the optimal dilution of the tested sera, positive and negative sera were diluted to generate five serial dilutions (1:50, 1:100, 1:200, 1:400, and 1:800).

### 2.9. Comparative Analysis of the Clinical Samples

A total of 208 clinical serum samples were collected from Inner Mongolia, China in 2022 and stored in our laboratory. In order to perform comparative analysis of the clinical samples, the novel cELISA, the commercial cELISA kit (VMRD, Pullman, WA, USA) and the western blot were used simultaneously to detect the antibodies against *T. equi* and *B. caballi*. The Venn diagram shows the comparative results of the novel cELISA method, the western blotting method and the commercial cELISA method for the diagnosis of clinical samples of *T. equi* and *B. caballi*. Venn plot map analyses were performed using the Jvenn tool, a free online platform (http://www.bioinformatics.com.cn (accessed on 28 October 2022)) for data analysis [22].

## 3. Results

### 3.1. Preparation of the Proteins EMA1 and Bc48 and the Corresponding HRP Labeled mAbs

SDS-PAGE verification showed that the EMA1 and Bc48 proteins were successfully expressed using the aforementioned prokaryotic expression system for the production of proteins of the expected sizes of 42 kDa and 40 kDa, respectively [21]. Finally, 20 mg (1 mg/mL) of purified recombinant EMA1 protein and 20 mg (1 mg/mL) of purified recombinant Bc48 protein were obtained, respectively. The correctly identified EMA1 and Bc48 proteins were stored at −80 °C. Next, the purified proteins were used as antigens for the immunization of mice to generate mAbs for use in cELISA development. The specificity of 1A12 (EMA1) and 1A3 (Bc48) were further confirmed by results of competitive binding assays, designed to detect the capability of immune sera of known specificity to inhibit the binding of each mAb to its respective antigens. Hybridoma cells that could secrete mAbs against EMA1 or BC48 were collected and mAbs clearly reacted with the recombinant EMA1 and Bc48 proteins. After identification by using an antibody subtype kit, mAbs 1A12 and 1A3 were both determined to be IgG1 subtypes. Then, 15 mg (1 mg/mL) of the purified mAb 1A12 and 15 mg (1 mg/mL) of the purified mAb 1A3 were obtained. Both mAbs 1A12 and 1A3 were then labeled with HRP using the HRP-labeling kit, and 5 mg of each HRP-labeled mAb was stored in −80 °C refrigerator with 50% glycerol.

### 3.2. Establishment of the cELISA Method

**Figure 1:**
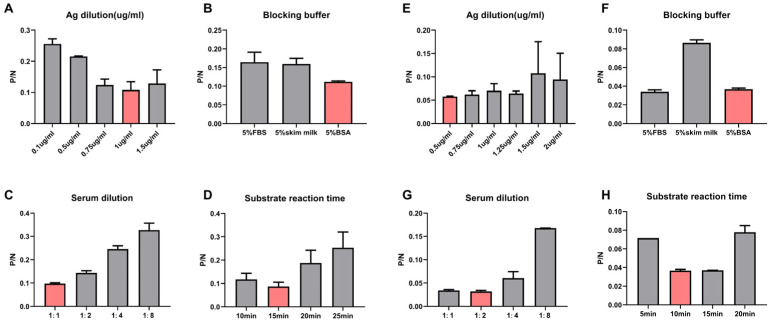
Determination of optimal antigene dilution, blocking buffer, serum dilution, and substrate reaction time. Determination of concentrations of EMA1 protein (**A**), blocking buffer (**B**), serum dilution (**C**), and substrate reaction time (**D**) to be used in the novel cELISA to detect antibodies against *T. equi.* Determination of concentrations of Bc48 protein (**E**), blocking buffer (**F**), serum dilution (**G**), and substrate reaction time (**H**) to be used in the novel cELISA to detect antibodies against *B. caballi*.

The checkerboard titration method was used to optimize our cELISA diagnostic method for *T. equi* or *B. caballi*, separately [23]. The results showed that the optimal EMA1 protein coating concentration for the detection of *T. equi* was l µg/mL (Figure 1A), and the most suitable blocking buffer was 5% BSA (Figure 1B). The optimal dilution of the equine serum sample to be tested was 1:1 (Figure 1C). The optimal colorimetric reaction time was 15 min since the P/N value at this time was the minimum (Figure 1D).

The optimal coating concentration of Bc48 protein for the detection of *B. caballi* was 0.5 µg/mL (Figure 1E) and the most suitable blocking buffer was 5% BSA (Figure 1F). The optimal dilution of the equine serum sample to be tested was found to be 1:2 (Figure 1G). The optimal colorimetric reaction time was 10 min since the P/N value at this time was the minimum (Figure 1H).

### 3.3. Determination of the cELISA Cut-Off Value (PI)

**Figure 2:**
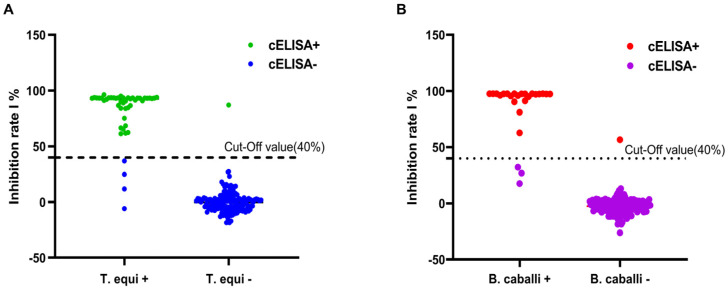
Determination of the cut-off value of the novel cELISA assays. Two hundred equine serum samples were assessed using the novel cELISA to determine the cut-off value. When the consistency and identification results of the serum samples by the novel cELISA methods were at their maximum, the cut-off values of both novel cELISA assays were found to be 40% (**A**,**B**).

Two hundred equine serum samples were assessed using our novel cELISA to determine the cut-off value of the cELISA. The samples were collected from Inner Mongolia from 2019 to 2020 and were kept in our laboratory. Of these samples, 47 were positive for *T. equi* and 153 were negative for *T. equi*, and 25 samples were positive for *B. caballi* and 175 were negative for *B. caballi*. Our results suggested that when the cut-off value was set to 40%, the consistency and identification results of the serum samples by the novel cELISA methods were at their maximum, so the cut-off value for both novel cELISA was 40.0% (Figure 2A,B). When the PI value of the tested serum sample was equal to or greater than 40%, it was determined to be antibody-positive and the sample was regarded to be antibody-negative at a PI < 40%.

### 3.4. Analysis of the Specificity of the Novel cELISA

**Figure 3:**
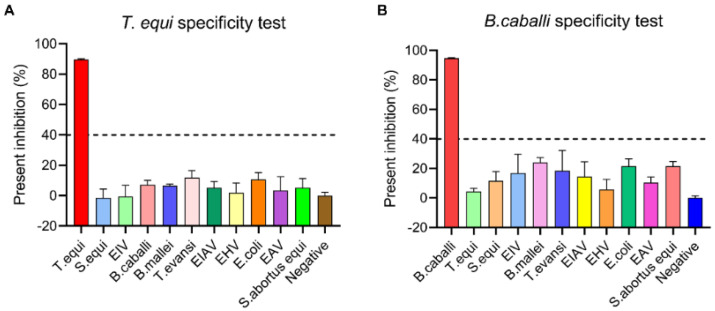
Analysis of the specificity of the novel cELISA. Cross-reaction of the novel cELISA to *T. equi* (**A**) or *B. caballi* (**B**). Sera positive for other pathogens, including EIAV, EAV, EHV-4, EIV, *B. caballi*, *B. mallei*, *T. equi T. evansi*, *E. coli*, *S. equi*, *S. abortus equi*, and a negative sample, were tested with the novel cELISA at the same time.

To determine the analytical specificity of detection of *T. equi* antibodies, sera positive for other common equine diseases, including EIAV, EAV, EHV-4, EIV, *B. caballi*, *B. mallei*, *T. evansi*, *E. coli*, *S. equi*, and *S. abortus equi*, were assessed using the cELISA to determine cross-reactivity. As shown in Figure 3A, the resulting PI values for *T. equi* antibody-positive sera were higher than 40%, while the PI values of sera positive for other equine diseases were all under 40%, and were therefore assessed as negative. Similarly, when the cELISA was tested for specificity for antibodies against *B. caballi* as well as other common equine diseases (EIAV, EAV, EHV-4, EIV, *T. equi*, *B. mallei*, *T. evansi*, *E. coli*, *S. equi*, and *S. abortus equi*), the PI values of the serum samples positive for other common equine diseases were all lower than 40%, and therefore tested as negative, while those positive for *B. caballi* were above 40% and therefore considered to be positive (Figure 3B). These results indicated that the novel cELISA assays do not detect antibodies against pathogens except for *T. equi* or *B. caballi* in equine sera, and the tests, therefore, have high specificity.

### 3.5. Analysis of the Sensitivity of the Novel cELISA

**Figure 4:**
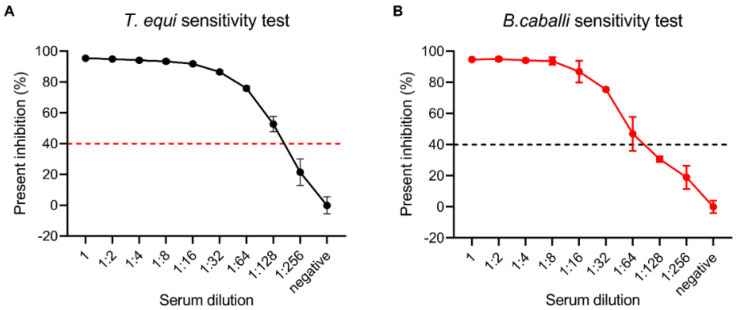
Analysis of the sensitivity of the novel cELISA. Serial two-fold dilutions of positive sera and a negative serum sample were tested with the novel cELISA, and percent inhibition was recorded. (**A**) Performance of the novel cELISA when detecting antibodies against *T. equi* in *T. equi*-positive sera at 0:1, 1:2, 1:4, 1:8, 1:16, 1:32, 1:64, 1:128, and 1:256 dilutions, and a negative *T. equi* serum sample. (**B**) Performance of the novel cELISA when detecting antibodies against *B. caballi* in *B. caballi*-positive sera at 0:1, 1:2, 1:4, 1:8, 1:16, 1:32, 1:64, 1:128, and 1:256 dilutions, and a negative *B. caballi* serum sample.

Different dilutions of *T. equi*-positive serums were used to determine the analytical sensitivity of the novel cELISA. The results showed that the PI was greater than 40% when the *T. equi*-positive serum was diluted 1:128 and was less than 40% when the *T. equi*-positive serum was diluted 1:256 (Figure 4A). Similarly, the analytical sensitivity of the novel cELISA for *B. caballi* was determined using different dilutions of *B. caballi*-positive serums. The results showed that the PI was greater than 40% when the *B. caballi*-positive serum was diluted 1:64 and was less than 40% when the *B. caballi*-positive serum was diluted 1:128 (Figure 4B). So, the data indicated the analytical sensitivity of the novel cELISA for *T. equi* and *B. caballi* were 1:128 and 1:64, respectively. Overall, the developed novel cELISA methods exhibit good sensitivity and have promise as candidates for a commercial kit.

### 3.6. Comparison of the Novel cELISA and the Commercially Available cELISA Kit

**Figure 5:**
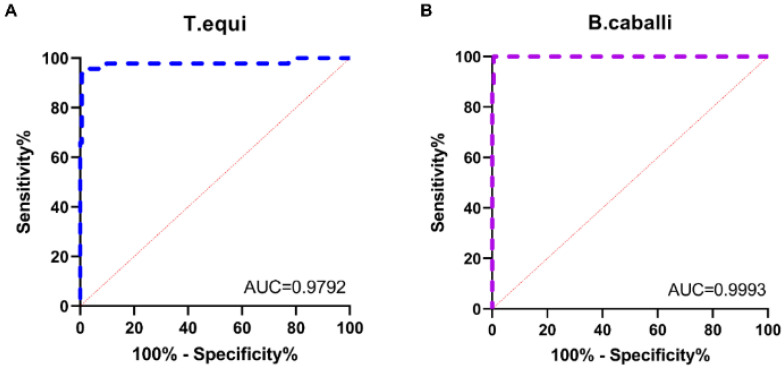
ROC curve analysis of the novel cELISA assay for clinical sample testing. The ROC curve was plotted by calculating the diagnostic sensitivity and specificity of the novel cELISA PI values relative to the results of the commercial cELISA assay (**A**,**B**).

To evaluate the performance of this novel cELISA assay, a total of 200 serum samples were tested using the novel cELISA and a commercially available cELISA kit. All samples were tested with the novel cELISA, as well as the commercially available cELISA kit, and the PI value of each sample was determined. We performed receiver operating characteristic (ROC) curve analysis as a statistical tool for the diagnostic evaluation of the novel cELISA in surveillance samples, where the area under the ROC curve (AUC) was employed to assess the accuracy of the novel cELISA assays (an AUC of 1 indicates perfect discriminatory value; an AUC of 0.5 or less indicates no discriminatory value). Compared with the commercial cELISA method, the AUCs of the novel cELISA assay were 0.979 for *T. equi* and 0.999 for *B. caballi* (*p* < 0.001) (Figure 5A,B), and the diagnostic sensitivity and specificity of our novel cELISA assay were 91.49% (43/47) and 99.35% (152/153) for *T. equi*, and 88% (22/25) and 99.43% (174/175) for *B. caballi* (Figure 5A,B). The agreement among the assays was calculated as previously reported [24], and the coincidence rate between the novel cELISA and the commercial cELISA assays was 97.5% for *T. equi* and 98% for *B. caballi*.

### 3.7. Establishment of the Western Blot Immunoassay

**Figure 6:**
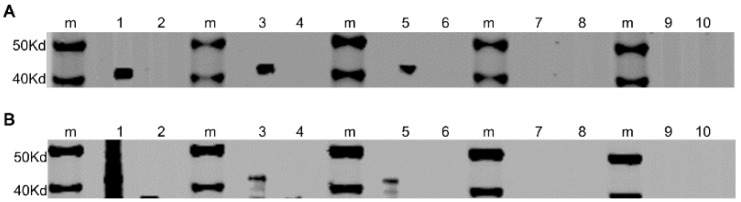
Determination of the optimum dilution of the test sera for western blot immunoassay. Lines: m, maker; 1, 1:50 (positive sera); 2, 1:50 (negative sera); 3, 1:100 (positive sera); 4, 1:100 (negative sera); 5, 1:200 (positive sera); 6, 1:200 (negative sera); 7, 1:400 (positive sera); 8, 1:400 (negative sera); 9, 1:800 (positive sera); 10, 1:800 (negative sera). (**A**) The determination of the optimum dilution of the test sera for the western blot assay for *T. equi* detection. (**B**) The determination of the optimum dilution of the test sera for the western blot assay for *B. caballi* detection.

To determine the optimum dilution of the test sera for the novel western blot immunoassay, we assessed five serial dilutions of the serum samples either negative or positive for *T. equi* or *B. caballi* using the established western blot assay. The results showed that the optimum serum dilution in the western blot assay of antibodies against *T. equi* or *B. caballi* was determined to be 1: 200 (Figure 6A,B). Under these conditions, *T. equi* or *B. caballi*-positive sera showed the target band, while the sera negative for these diseases did not have the target band.

To determine the optimum dilution of the test sera for the novel western blot immunoassay, we assessed five serial dilutions of the serum samples either negative or positive for *T. equi* or *B. caballi* using the established western blot assay. The results showed that the optimum serum dilution in the western blot assay of antibodies against *T. equi* or *B. caballi* was determined to be 1: 200 (Figure 6A,B). Under these conditions, *T. equi* or *B. caballi*-positive sera showed the target band, while the sera negative for these diseases did not have the target band.

### 3.8. Clinical Performance

For evaluating whether the novel cELISA and the western blot assay can be used to accurately detect an instance of disease in clinical samples, a total of 208 serum samples collected in Inner Mongolia, China in 2022 were assessed using the novel cELISA, the commercially available cELISA kit, and the western blot assays. Venn diagrams were used to compare the performance of the different assays in detecting antibodies against *T. equi* or *B. caballi.*

**Figure 7:**
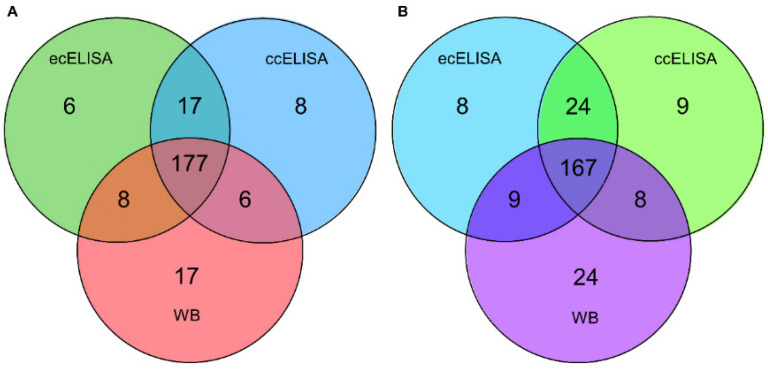
Venn diagram showing summary results of serological detection methods (newly established cELISA (ecELISA), commercial cELISA (ccELISA), and western blot (WB) assays) for serum samples from equids. A total of 208 serum samples collected in Inner Mongolia, China in 2022 were included in the comparison. (**A**) Venn diagrams were used to compare the performance of the different assays in detecting antibodies against *T. equi.* (**B**) Venn diagrams were used to compare the performance of the different assays in detecting antibodies against *B. caballi*.

The three methods of detection agreed in 177 out of the total 208 serum samples tested for the presence of *T. equi* antibodies (Figure 7A). Across all 208 samples analyzed for the presence of *T. equi* antibodies, coincidence rates of the novel cELISA with the western blot assay and commercial cELISA assay were 93.27% (194/208) and 87.98% (183/208), respectively.

The three methods of detection agreed in 167 out of the total 208 serum samples tested for the presence of *B. caballi* antibodies. The coincidence rate between the novel cELISA and the commercial cELISA was 91.83% (191/208), and that between the western blot assay and the commercial cELISA was 84.13% (175/208). Collectively, these results suggest that the novel cELISA and the western blot assay have a high agreement with the commercial kit, and they are promising candidates for further clinical testing.

## 4. Discussion

EP has emerged as an important protozoan infection and is caused by *T. equi* and *B. caballi* [25]. A successful vaccine against EP has yet to be developed, and the significant genetic diversity within *T. equi* and *B. caballi* clades complicates vaccine development efforts. The economic impact of this disease and the restrictions on the trade of infected animals have led the WOAH Animal Health Code to categorize EP as a notifiable disease [26]. This highlights the need for robust and effective measures of control against the disease in countries where it is endemic, and for combined high-sensitivity serological techniques to enhance the detection of the disease in animals exposed to the parasites that cause EP. The current techniques for diagnosing *T. equi* and *B. caballi* infections include microscopic examination, CFT, IFAT, cELISA, and PCR [27]. Currently, the WOAH considers cELISA to be the preferred test for EP in the international horse trade [7]. The cELISA method overcomes problems of antigen purity since the specificity of the cELISA depends solely on the mAb used. For this reason, the cELISA method is ideal for detection with recombinant antigens. The use of recombinant protein in diagnostic assays also precludes the need to infect animals or cells with the pathogen for antigen production [28]. To prevent the further spread of the infection, some countries (the USA, Canada, Australia, and Japan) require verification of the seronegativity of imported horses [29]. The commercial cELISA kit that allows testing for EP is relatively time-consuming and requires complex procedures. However, there is no developed commercial test kit for EP in China. This makes the control of this disease in China greatly challenging. This study aimed to establish cELISA methods for the detection of EP. In order to improve the accuracy of EP diagnosis, the presence of cross-reactions and potential performance issues of the several assays available should always be considered when interpreting laboratory results. Therefore, a western blotting method was established as a supplementary method for the diagnosis of *T. equi* and *B. caballi*.

Rapid and sensitive detection methods are critical for performing epidemiological investigations and for disease control and prevention. ELISA is therefore a good choice in the large-scale monitoring of disease outbreaks. Due to their low cost and high throughput, ELISA assays have been widely used for the serological diagnosis of *T. equi* and *B. caballi* [30]. However, the design of some commercial cELISA kits relies on species-specific HRP-labeled secondary antibodies [18]. The difference between the novel cELISA and the commercial cELISA is that the novel cELISA directly uses HRP-labeled mAbs as the detection antibody, while the commercial cELISA uses HRP-labeled anti-horse IgG secondary antibody as the detection antibody. The advantages of using competitive mAbs for detecting antibodies against *T. equi* and *B. caballi* include the reduced number of operation steps, a reduction in the testing time, and unlimited provision of a standardized reagent. In addition, the detection results were made more specific by reducing the operational step of one-step cascade amplification. The proteins EMA1 and Bc48 are commonly used as diagnostic antigens against *T. equi* and *B. caballi*, respectively. In this study, HRP-labeled 1A12 and 1A3 mAbs were used as detection antibodies to establish the cELISA assay. The results showed that the novel cELISA had high sensitivity and specificity (Figure 3 and Figure 4), and had a high coincidence rate with the commercial cELISA assay (Figure 5).

Western blotting is used as a supplementary diagnostic test for important human and veterinary diseases, including human immunodeficiency virus (HIV), transmissible spongiform encephalopathy (TSE), and equine infectious anemia virus (EIAV) [31,32,33]. In this study, the newly established western blotting detection method showed high consistency with the results of the commercial cELISA kit from VMRD (Figure 7A,B). A potential explanation for this discrepancy observed in some clinical samples is that the native structure of EMA1 and Bc48 proteins and recombinant EMA1 and Bc48 proteins were different. In conclusion, we have defined the most important quality parameters and have found that the performance of our western blot assay is sufficient to make it a reliable diagnostic tool to be used as a follow-up test after a doubtful ELISA test result. This tool is particularly relevant because it represents a new method to confirm discordant or inconclusive test results from cELISA. Following more extensive validation, the use of this assay in screening horses prior to inter-country movement may help with the continued control of EP on a global scale.

Overall, this study has established novel cELISA and western blotting methods for the detection and diagnosis of EP. The novel cELISA and western blotting techniques showed comparable sensitivity and specificity to the commercially available cELISA. We suggest the use of western blot assay as a complementary test, available for the confirmation of conflicting results, that will contribute to resolving legal and/or sanitary situations that could lead to the spread of EP in China.

## Data Availability

Not applicable.
